# Global DNA Hypomethylation Is Associated with High Serum-Persistent Organic Pollutants in Greenlandic Inuit

**DOI:** 10.1289/ehp.11338

**Published:** 2008-07-16

**Authors:** Jennifer A. Rusiecki, Andrea Baccarelli, Valentina Bollati, Letizia Tarantini, Lee E. Moore, Eva C. Bonefeld-Jorgensen

**Affiliations:** 1 Department of Preventive Medicine and Biometrics, Uniformed Services University of the Health Sciences, Bethesda, Maryland, USA; 2 Department of Environmental and Occupational Health, University of Milan and IRCCS Maggiore Hospital, Regina Elena and Mangiagalli, Center of Molecular and Genetic Epidemiology, Milan, Italy; 3 Division of Cancer Epidemiology and Genetics, Occupational and Environmental Epidemiology Branch, National Cancer Institute, National Institutes of Health, Department of Health and Human Services, Bethesda, Maryland, USA; 4 University of Aarhus, Unit of Cellular and Molecular Toxicology, Centre of Arctic Environmental Medicine, Institute of Public Health, University of Aarhus, Aarhus, Denmark

**Keywords:** DNA methylation, global methylation, Greenland, hypomethylation, Inuit, organochlorines, PCBs, persistent organic pollutants, pesticides, polychlorinated biphenyls, POPs, serum

## Abstract

**Background:**

Persistent organic pollutants (POPs) may influence epigenetic mechanisms; therefore, they could affect chromosomal stability and gene expression. DNA methylation, an epigenetic mechanism, has been associated with cancer initiation and progression. Greenlandic Inuit have some of the highest reported POP levels worldwide.

**Objective:**

Our aim in this study was to evaluate the relationship between plasma POPs concentrations and global DNA methylation (percent 5-methylcytosine) in DNA extracted from blood samples from 70 Greenlandic Inuit. Blood samples were collected under the Arctic Monitoring and Assessment Program and previously analyzed for a battery of POPs.

**Methods:**

We used pyrosequencing to estimate global DNA methylation via *Alu* and LINE-1 assays of bisulfite-treated DNA. We investigated correlations between plasma POP concentrations and global DNA methylation via correlation coefficients and linear regression analyses.

**Results:**

We found inverse correlations between percents methylcytosine and many of the POP concentrations measured. Linear regressions, adjusting for age and cigarette smoking, showed statistically significant inverse linear relationships mainly for the *Alu* assay for *p*,*p*′-DDT (1,1,1-trichloro-2,2-bis(*p*-chlorophenyl)ethane; β = −0.26), *p*,*p*′-DDE [1,1-dichloro-2,2-bis(*p*-chlorophenyl) ethylene; β = −0.38], β-hexachlorocyclohexane (β = −0.48), oxychlordane (β = −0.32), α-chlordane (β = −0.75), mirex (β = −0.27), sum of polychlorinated biphenyls (β = −0.56), and sum of all POPs (β = −0.48). Linear regressions for the LINE-1 assay showed β estimates of similar magnitudes to those using the *Alu* assay, however, none was statistically significant.

**Conclusions:**

This is the first study to investigate environmental exposure to POPs and DNA methylation levels in a human population. Global methylation levels were inversely associated with blood plasma levels for several POPs and merit further investigation.

Greenlandic Inuit are highly exposed to persistent organic pollutants (POPs) from their diet and from the contamination of the Arctic environment from emissions at lower latitudes (Deutch and Hansen 2000). Some of the highest biologic concentrations of POPs measured in humans worldwide have been among Greenlandic Inuit (Danish Environmental Protection Agency 1997; Dewailly et al. 1999). There is some evidence from human studies that exposure to POPs, many of which are known endocrine disruptors, may increase the risk of certain cancers, such as non-Hodgkin lymphoma (Colt et al. 2005; Engel et al. 2007), pancreatic cancer (Hardell et al. 2007; Hoppin et al. 2000; Porta et al. 1999), and prostate cancer (Ritchie et al. 2003). In addition, POP exposures have been associated with autoimmune diseases (Powell et al. 1999), diabetes, (Longnecker and Daniels 2001), developmental neurotoxicity (Kakeyama and Tohyama 2003; Seegal 1996; Tilson and Kodavanti 1998; Winneke et al. 2002), and adverse reproductive end points, such as spontaneous abortion (Korrick et al. 2001; Longnecker et al. 2005; Venners et al. 2005), birth defects (Salazar-Garcia et al. 2004), and impaired male fertility (Cocco et al. 2005; Pflieger-Bruss et al. 2004). However, the mechanisms by which POPs can lead to these diseases and adverse outcomes are not well characterized. We hypothesized that POPs may operate at the epigenetic level by influencing chromosomal stability and gene expression and not by directly affecting changes in DNA sequence. Aberrant DNA methylation is an epigenetic mechanism that has been associated with a wide range of diseases, including those potentially associated with exposure to POPs (Balada et al. 2007; Perrin et al. 2007; Wilson et al. 2007). In particular, global DNA hypomethylation is generally associated with chromosomal instability, reactivation of retrotransposable elements, and the expression of genes that would normally be silenced by methylation (Jones and Baylin 2002; Schulz 2006; Smith and Crocitto 1999).

To investigate whether these POPs may operate at the epigenetic level and to test the hypothesis that POPs exposure is associated with global DNA hypomethylation (i.e., an inverse association with DNA methylation), we examined the correlation between lifetime body burden of POPs and percentage of global genomic DNA methylation, percent 5-methylcytosine (%5-mC), in a highly exposed population. Blood specimens had been collected previously from Greenlandic Inuit (*n* = 71)and analyzed for a battery of POPs (Deutch and Hansen 2000). For this study, DNA was extracted from the same whole blood samples, and we investigated global genomic DNA in repetitive elements *Alu* and LINE-1 (long interspersed nucleotide element). Because there are approximately 1.4 million *Alu* repetitive elements in the human genome and a half a million LINE-1 elements (Kazazian and Goodier 2002; Yang et al. 2004) that are normally heavily methylated, and it is estimated that more than one-third of DNA methylation occurs in repetitive elements, the %5-mC of repetitive elements can act as a surrogate marker for percentage of global genomic DNA methylation (Yang et al. 2004).

## Methods

### Study population and sample collection and processing

Blood samples were previously collected from 71 Greenlandic Inuit under the Arctic Monitoring and Assessment Program (AMAP), a working group tasked by the Arctic Council to monitor the levels of pollutants and to assess the effects of pollution in all compartments of the Arctic environment, including human populations. The study population and protocol were described previously (Deutch and Hansen 2000). Briefly, participants in the study were consecutive general practice patients recruited by local district hospitals during a period of 1–2 months. Participants were from various towns throughout Greenland, representing different geographical regions: from Scoresbysund (central east), Tasiilaq (south east), Nanortalik (south west), Nuuk (south west), Ilulissat (central sest), and Upernavik (central west). Blood draws were taken and demographic/lifestyle data were collected from September 1997 to April 1998. All participants provided informed written consent, and the institutional review boards of the Uniformed Services University approved the protocol.

Blood samples were collected at the local district hospitals, separated into plasma and ETDA whole blood, frozen at −20°C, and transported to Nuuk, where they were stored at −80°C until further transport to the Department of Environmental and Occupational Medicine, Aarhus University. Using gas chromatography (GC) as described previously (Butler Walker et al. 2003) plasma samples (2 mL) were analyzed at Le Centre de Toxicologie (Sainte Foy, Quebec, Canada) for the following POPs: *p*,*p*′-DDT [1,1,1-trichloro-2,2-bis(*p*-chlorophenyl)ethane], *p*,*p*′-DDE [1,1-dichloro-2,2-bis(*p*-chlorophenyl) ethylene], β-hexachlorocyclohexane (β-HCH), hexachlorobenzene, chlordane, *cis*-chlordane, oxychlordane, α-chlordane, mirex, toxaphene, and polychlorinated biphenyl (PCB) congeners 28, 52, 99, 101, 105, 118, 128, 138, 153, 156, 170, 180, 183, and 187. The methods have been described previously (Deutch and Hansen 2000). The recovery of congeners using parallel plasma samples spiked with ^13^C- or ^14^C-PCB standards was calculated to 98–100%. We determined plasma lipid levels for total cholesterol (TC), free cholesterol (FC), triglycerides (TG), and phospholipids (PL) as well as total lipids using the following calculation: total lipids = 1.677 (TC − FC) + FC + TG + PL (Patterson et al. 1991). All plasma POPs concentrations were adjusted for lipids.

For 70 of the 71 samples, DNA was isolated from EDTA-whole blood at the University of Aarhus. Cells were lyzed by a solution of 10% Triton-X, 0.32 M sucrose, 5 nM MgCl_2_, and 1 mM Tris-HCl (pH 7.5). After centrifugation at 4,000 rpm (4°C), the proteins and RNA were cleared away in a solution of proteinase K (2 mg/mL), 400 mM NaCl, 2 mM EDTA (pH 8.0), 10 mM Tris-HCl (pH 7.5), and 5% sodium dodecyl sulfate, followed by overnight incubation at 37°C. Then 27% saturated NaCl was added, mixed vigorously, and centrifuged at 6,000 rpm for 15 min; the DNA was then isolated from the supernatant using 96% ethanol at −20°C overnight. The DNA pellet was resuspended in 200 mL 1 mM Tris-EDTA buffer (pH 8.0) and stored at 4°C.

### Bisulfite treatment

We treated 1 μg DNA (concentration 50 ng/μL) using the EZ DNA Methylation-Gold Kit (Zymo Research, Orange, CA, USA) according to the manufacturer’s protocol. Final elution was performed with 30 μL M-Elution Buffer (Zymo Research). Bisulfite-treated DNA was stored at −20°C until use.

We used a built-in control to verify bisulfite conversion efficiency by pyrosequencing: we used a C outside a CG site. After bisulfite treatment, the conversion of this C into T is expected to be 100%. It is possible to insert a C/T single-nucleotide polymorphism into the sequence to be analyzed that will result in a 100% T if conversion is efficient.

### Polymerase chain reaction (PCR) and pyrosequencing

We quantified DNA methylation (%5 mC) using PCR-pyrosequencing of the bisulfite-treated DNA, as previously described (Bollati et al. 2007). In brief, the bisulfite-treated samples were amplified by PCR. We then used a biotin-labeled primer to purify the final PCR product using Sepharose beads. The PCR product was bound to Streptavidin Sepharose HP (Amersham Biosciences, Uppsala, Sweden), and the Sepharose beads containing the immobilized PCR product were purified, washed, and denatured using a 0.2 M NaOH solution, and washed again using the Pyrosequencing Vacuum Prep Tool (Pyrosequencing, Inc., Westborough, MA, USA), as recommended by the manufacturer. Then 0.2 μM pyrosequencing primer was annealed to the purified single-stranded PCR product and pyrosequencing was performed using the PSQ-HS96 Pyrosequencing System (Pyrosequencing, Inc.). We used *Alu* and LINE-1 element PCR for pyrosequencing-based methylation analysis according to previously published methods (Yang et al. 2004), with the following modifications: a 50-μL PCR was carried out in 25-μL GoTaq Green Master mix (Promega, Madison, WI, USA), 1 pmol of the biotinylated forward primer, 1 pmol of the reverse primer, 50 ng of bisulfite-treated genomic DNA, and water.

The percentage of methylation was expressed for each DNA locus as %5-mC divided by the sum of methylated and unmethylated cytosines. We tested each marker in three replicates and used their average in the statistical analyses. Some controls were included in every pyrosequencing run. To ensure that there was no contamination, some wells were filled with water. To be sure that pyrosequencing was sequencing the correct pattern, one well was filled with an oligonucleotide with a known sequence. To control repeatability of the assay, we filled a well with fully methylated DNA and a well with unmethylated DNA.

### Statistical analyses

We calculated means and medians of each POP measured and mean ± SD %5-mC of Alu and LINE for our study population and various strata of the group, using a *t*-test to evaluate the difference in %5-mC between males and females, younger age (19–35 years) and older age (36–67 years), and smoking (≤ 5 cigarettes/ day, > 5 cigarettes/day).

Using a correlation coefficient (*r*) for each LINE-1 and *Alu* assay, we investigated the relationships between %5-mC and the lipid-adjusted concentrations of the tested POPs. We also used the sum of the 14 PCBs measured (∑PCBs) and the sum of all POPs measured (∑POPs). Because the distributions of the POPs were highly skewed to the right, we calculated a Pearson correlation coefficient using log-transformed POP concentrations; we also calculated a Spearman correlation coefficient.

We then carried out linear regression analyses, modeling each log-transformed POP concentration on %5-mC. Because both age (Fraga et al. 2007; Liu et al. 2003) and smoking (Furniss et al. 2008; Smith et al. 2007) have been found to be associated with global hypomethylation and because age is strongly correlated with POP levels, we adjusted for both as continuous variables (a level of zero was assigned to nonsmokers). We also attempted to further investigate potential effect modification from each of these variables by stratifying our models by median age (younger group, 19–35 years; older group, 36–67 years) and smoking level [low: ≤ 5 cigarettes/day (zero was included); high: > 5 cigarettes/day (range, > 5–35)] and carrying out a test for interaction. Although there is no *a priori* evidence that sex is associated with global hypomethylation, we wanted to investigate whether there were differences according to sex in our population. As statistical power was of concern due to the small sample size of this study, we did not adjust regression models by sex because the categories were so unequal. Thus, we carried out linear regression analyses on the total population (*n* = 70) and then stratified by sex to investigate potential differences. All statistical tests were two sided, and analyses were carried out using SAS software, version 9.1 (SAS Institute Inc., Cary, NC, USA).

## Results

Among the 70 people included in this study, 61 were male and 9 were females (all females were from the eastern town of Tasiilaq), and age ranged from 19 to 67 years. Eleven (15.7%) of the participants reported that they never smoked, 18 (25.7%) reported smoking 1–5 cigarettes/day, and 41 (58.6%) reported smoking 6–35 cigarettes/day. Mean values, medians, and ranges of each of the POPs measured are presented in Table 1.

The mean ± SD %5-mC was 25.26 ± 0.84 (range, 23.20–27.30) in the *Alu* assay and 78.88 ± 2.72 (range, 73.00–84.70) in the LINE-1 assay (Table 2). For both the *Alu* and LINE-1 assays, the distribution was fairly normal. The average %5-mC did not differ substantially between younger and older age groups or between lower- and higher-smoking groups. We did find a difference, however, when we applied a *t*-test for unequal variance between males and females for both the *Alu* assay (*p* < 0.0001) and the LINE-1 assay (*p* = 0.02); males had higher percent methylation than females. This finding could be due to the very small number of females in this study (*n* = 9), and therefore the estimate may be imprecise.

**Figure 1:**
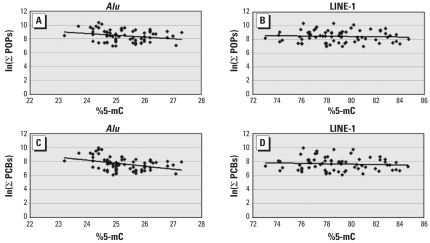
Linear plots of %5-mC, based on *Alu* and LINE-1, versus the natural log of ∑POPs and ∑PCBs. (*A) Alu* versus ln(∑POPs). (*B*) LINE-1 versus ln(∑POPs). (*C*) *Alu* versus ln(∑PCBs). (*D*) LINE-1 versus ln(∑PCBs).

Figure 1 presents scatterplots for *Alu* and LINE-1 %5-mC versus the log-transformed ∑POPs and ∑PCBs, which indicate a negative linear trend. This was confirmed by the inverse correlations (*r*) we found for both Pearson and Spearman coefficients between %5-mC and many of the POP (log-transformed) concentrations (i.e., with increasing concentration of POPs, there was a decreasing degree of DNA methylation); Table 3 presents results for the Pearson correlation coefficient only. The estimates for *r* for *Alu* were more strongly inverse (most in the range of −0.20 to −0.50) and many of these values were statistically significant compared with those for the LINE-1 assay, the estimates of which were generally closer to zero, and none was statistically significant. Restricting our analyses to male subjects only yielded similar results to those carried out for the whole population.

Linear regressions, adjusting for age and cigarette smoking, showed significant inverse linear relationships, again mainly for the *Alu* assay for the following POPs: *p*,*p*′-DDT (β = −0.26, *p* = 0.01), *p*,*p*′-DDE (β = −0.38, *p* = 0.01), β-BHC (β = −0.48, *p* < 0.01), oxychlordane (β = −0.32, *p* < 0.01), α-chlordane (β = −0.75, *p* = 0.05), mirex (β = −0.27, *p* = 0.01), sum of PCBs (β = −0.56, *p* < 0.01; there were significant inverse linear relationships for all PCB congeners), and sum of all POPs (β = −0.48, *p* < 0.01) (Table 4). Results for linear regressions using the LINE-1 assay generally showed βestimates of a similar magnitude to those using the *Alu* assay; however, none was statistically significant. There was a very strong, marginally significant negative linear relationship for the LINE-1 assay for β-BHC (β = −0.98; *p* = 0.06). When we stratified the population into males (*n* = 61) and females (*n* = 9) and ran the linear regressions above, the βcoefficients did not differ substantially.

We stratified by younger age and older age and by low smoking and high smoking. We found negative linear relationships between POPs and percent methylation for each of the age strata and smoking strata; however, the estimates were more strongly negative and statistically significant for the *Alu* assay (data not shown). We found no significant differences in the linear relationships between younger and older age groups and between low smoking and high smoking groups. Tests for interaction also indicated that neither of these variables is an effect modifier.

To investigate our findings further, we categorized each individual POP into tertiles and carried out linear regression analyses, comparing the second and third tertiles to the first tertile. The results from this analysis confirmed our findings of an inverse linear relationship (i.e., as found for the POPs in the model as a continuous variable), in that the β coefficient for the third tertile was more strongly inverse than the βcoefficient for the second tertile. For most POPs, estimates in the third tertile were statistically significant for the *Alu* assay but not the for the LINE-1 assay (data not shown)

## Discussion

*In vitro* and animal studies have suggested that exposure to endocrine disruptors, such as POPs, may adversely affect DNA methylation patterns (Anway et al. 2006; Anway and Skinner 2006; Chang et al. 2006; Kang and Lee 2005). To our knowledge, this had not previously been investigated in a human population. We observed a consistent inverse correlation and linear relationship between POPs levels and percent methylation of *Alu* and LINE-1 repetitive elements, though the results were more marked and statistically significant for the *Alu* assay. LINE-1 and *Alu* are controlled through different mechanisms (Gonzalgo and Jones 1997) and have different transcription patterns in response to cellular stressors (Li and Schmid 2001), so this could account for the differences we saw in magnitude and significance of correlations. We used DNA methylation analyses of *Alu* and LINE-1 repetitive elements to evaluate global methylation. Due to the heavy methylation of repetitive elements, these assays, which are easier to carry out than previous methods used to quantify total genomic 5-mC, can detect decreases in DNA methylation and serve as a surrogate for global methylation (Yang et al. 2004). Our results suggest that global hypomethylation is associated with high levels of environmental exposure to POPs. We must consider that the POPs investigated here are all correlated with each other, so it is not surprising that our findings of inverse correlations are consistent across POPs.

Compared to other populations described in a recent study (Rusiecki et al. 2005), the POP levels detected in this population are relatively high. For the organochlorine pesticides, such as *p*,*p*′-DDT, its metabolite *p*,*p*′-DDE, and β-HCH, the plasma levels observed in this population rank among the highest in the world. For individual PCB congeners, such as PCB-153 and PCB-180, the levels are comparable to those from another study among Inuit living in Nunavik, Canada (AMAP 2003; Ayotte et al. 1997). However, our study population is also characterized by a variation in concentrations, enabling us to fully investigate DNA methylation across a wide range.

We considered age an important confounder to control, because it is strongly correlated with serum POP concentrations and because epigenetic alterations, including global hypomethylation, can be progressively accumulated during aging and directly contribute to cell transformation (Fraga et al. 2007). In the present study, we detected the typical pattern of a positive correlation between age and almost all POPs we investigated (*r* values ranged from 0.22 to 0.65 and all were statistically significant at *p* ≤ 0.05). Despite the wide age range (19–67 years), which could reduce statistical power, and although the correlation between age and degree of DNA methylation using the *Alu* (*r* = 0.05, *p* = 0.60) and LINE-1 (*r* = 0.03, *p* = 0.75) assays was nonsignificant, we adjusted for age in our models and carried out stratified analyses by age. We made this adjustment because of *a priori* evidence of an association with both %5-mC and with POPs (Fraga et al. 2007). Smoking is also important to consider because it has been associated with a state of hypomethylation in various cancers (Furniss et al. 2008; Smith et al. 2007), so we included it in our final models, despite our *t*-tests not showing a difference in %5-mC between the two smoking groups. Similar to age, stratified analyses did not reveal differences between the two smoking groups. For neither age nor smoking did we find statistically significant interactions; however, our power was limited based on the small sample size of the study, especially after stratification, so we were limited in our ability to evaluate interaction. Regarding sex, although we found a significant difference between males and females with respect to %5-mC for both the *Alu* and LINE-1 assays, we did not find that the association between DNA methylation and POPs differed when we stratified linear regression analyses on sex. Again, we were likely inhibited in our ability to fully examine a potential sex difference because of the small sample size of the overall population and because there were only nine women included.

The biologic mechanism for POPs interfering with DNA methylation is unclear. Many of these POPs behave as endocrine disruptors, which may promote an alteration in DNA methylation sequences; this is associated with the development of transgenerational disease states (Chang et al. 2006). Recently, two endocrine disruptors, methoxychlor and vinclozolin, were shown to alter the spermatogenic capacity of male germ cells and sperm viability via their effects on DNA methylation (Anway and Skinner 2006; Tabb and Blumberg 2006). Another recent study found that MCF7 cells treated with butyl benzylphthalate, an endocrine disruptor due to its antiandrogenic or proestrogenic effects, altered estrogen receptor (ER) mRNA expression, and this may be related to aberrant DNA methylation in the promoter region of ER (Kang and Lee 2005).

Because of the cross sectional nature of the study, we were unable to determine if POP exposure leads to an aberrant global methylation status. In addition, in spite of the strong negative correlations and linear relationships, we cannot exclude that other contaminants ubiquitous in the Arctic environment, such as heavy metals (mercury, cadmium, lead) and radioactive elements (AMAP 2002) may have contributed to these findings. We did not have detailed smoking data, only the number of cigarettes smoked per day, which thus decreased our ability to evaluate smoking as an effect modifier. Another limitation of this study is that we did not have detailed dietary information, which could shed light on the accumulation of POP body burden over time and also could be important regarding folate intake. Folate plays an important role in DNA methylation: It is required for the synthesis of *S*-adenosyl methionine, the common methyl donor required for the maintenance of methylation patterns in DNA (Brunaud et al. 2003). Folate also plays a critical role in the prevention of chromosomal breakage and hypomethylation of DNA (Fenech 2001; Pyathilake and Johanning 2002). We carried out multiple comparisons in this study; however, because the majority of POPs showed a statistically significant inverse linear relationship in the *Alu* assay, our focus was on the detected pattern of association between degree of global methylation and lifetime POP body burden, rather than an association found for a specific POP.

A major strength of our study is that measurement of percent DNA methylation was based on a quantitative analysis using pyrosequencing methodology, which is highly reproducible and accurate for measuring small changes in DNA methylation. Epigenetic changes from environmental exposures or those occurring in the early stages of a disease such as cancer may not be as dramatic as those occurring in later stages; thus a quantitative measure of DNA methylation may be the only tool sensitive enough to elucidate such changes. Additionally, using pyrosequencing to quantify %5-mC enabled us to have a built-in quality control for bisulfite treatment (Dupont et al. 2004). The bisulfite treatment control is dependent on the fact that any cytosines not within a CpG site should be fully converted to thymines because these cytosines are not methylated. If the bisulfite treatment is not complete, the pyrosequencing reaction can detect the ratio of thymines to cytosines, thus indicating the efficiency of bisulfite treatment. Similarly, the ratio of thymines to cytosines at a particular CpG site after bisulfite treatment and PCR indicates the degree of methylation.

The present study shows for the first time that there is a relatively strong correlation between increasing serum levels of POPs and global DNA hypomethylation, which is an aberrant epigenetic pattern in malignant cells. These results support the hypothesis that exposure to potentially carcinogenic compounds such as POPs may influence DNA methylation. Our study was characterized by quantitative, biologic exposure assessment of lipid-adjusted, lifetime POP body burden in a population with some of the highest levels in the world and a highly reproducible, high throughput method to measure percent DNA methylation. The findings from this study are intriguing and merit further investigation.

## Figures and Tables

**Table 1:** Serum concentrations (ppb: ng/g-lipid) of POPs measured in 70 Inuit from various geographic regions of Greenland.

Chemical	No.	Mean	Median	Range
*p*,*p*’-DDT	70	44.03	25.08	4.04–372.88
*p*,*p*’-DDE	70	1624.1	1268.49	264.15–5968.66
β-HCH	70	56.75	39.98	6.25–290.32
Hexachlorobenzene	70	284.49	239.33	39.06–1000
Chlordane	70	1179.03	802.34	86.21–9485.94
*cis*-Chlordane	61	99.79	83.02	9.09–285.71
Oxychlordane	70	419.5	248.98	29.31–2419.35
α-Chlordane	62	1.69	1.57	1–5.8
Mirex	70	69.89	38.85	3.48–328.36
Toxaphene	70	230.75	193.78	15.91–773.95
PCB-28	70	7.44	5.41	1.14–39.56
PCB-52	70	12.35	8.96	1.06–82.61
PCB-99	70	120.65	92.52	15.38–532.26
PCB-101	70	11.87	8.54	1.28–69.57
PCB-105	70	24.39	13.98	1.82–161.29
PCB-118	70	117.71	78.46	13.64–693.55
PCB-128	70	4.04	2.96	1.12–14.52
PCB-138	70	572.5	412.86	93.59–3064.52
PCB-153	70	1135.78	687.06	152.83–7096.77
PCB-156	70	78.25	54.32	13.21–483.87
PCB-170	70	321.92	137.75	29.31–2419.35
PCB-180	70	697.36	318.43	66.04–5000
PCB-183	70	47.79	38.97	8.82–209.68
PCB-187	70	179.31	140.78	32.08–677.42
∑PCBs	70	3337.08	1991.06	449.06–20400
∑POPs	70	6826.13	4744.10	1035.85–31462.50

**Table 2:** Percent global DNA methylation (5-mC%) in *Alu* and LINE-1 markers in Greenlandic Inuit, whole population and various characteristics.

		*Alu* assay			LINE-1 assay		
Characteristic	No.	Mean	SD	*p*-Value^a^	Mean	SD	*p*-Value^a^
Whole population	70	25.26	0.84		78.88	2.72	
Sex							
Male	61	25.35	0.86		79.05	2.85	
Female	9	24.69	0.06	< 0.0001	77.73	1.18	0.02
Age group (years)							
Younger (19–35)	35	25.18	0.83		78.68	2.92	
Older (36–67)	35	25.34	0.84	0.44	79.09	2.53	0.53
Smoking							
Low (≤5 cigarettes/day)^b^	29	25.22	0.83		79.10	2.75	
High (> 5 cigarettes/day)	41	25.29	0.85	0.76	78.73	2.72	0.59

a *t*-test to examine differences in *Alu* and LINE for males vs. females, younger age vs. older age, and low vs. high smoking.

b Includes 0 cigarettes/day.

**Table 3:** Pearson correlation coefficients (*r*) for %5-mC and POPs concentrations (log-transformed) in serum from 70 Greenlandic Inuit.

	Mean *Alu*		Mean LINE-1	
Chemical	*r*	*p*-Value	*r*	*p*-Value
*p*,*p*′-DDT	−0.23	0.06	0.04	0.74
*p*,*p*′-DDE	−0.17	0.15	0.04	0.75
β-HCH	−0.19	0.11	−0.13	0.30
Hexachlorobenzene	0.15	0.20	−0.07	0.55
Chlordane	−0.04	0.73	−0.06	0.63
*cis*-Chlordane	0.10	0.43	0.00	0.99
Oxychlordane	−0.15	0.22	−0.12	0.33
α-Chlordane	−0.26	0.04*	−0.08	0.56
Mirex	−0.17	0.17	−0.03	0.82
Toxaphene	0.17	0.16	0.01	0.96
PCB-28	−0.22	0.07	−0.08	0.48
PCB-52	−0.07	0.59	0.00	0.98
PCB-99	−0.31	0.01*	−0.09	0.44
PCB-101	−0.15	0.20	0.11	0.35
PCB-105	−0.32	0.01*	−0.14	0.26
PCB-118	−0.23	0.05*	−0.11	0.37
PCB-128	−0.32	0.01*	0.02	0.88
PCB-138	−0.31	0.01*	−0.08	0.51
PCB-153	−0.38	< 0.01*	−0.09	0.44
PCB-156	−0.35	< 0.01*	−0.06	0.59
PCB-170	−0.49	< 0.01*	−0.12	0.32
PCB-180	−0.43	< 0.01*	−0.09	0.48
PCB-183	−0.35	< 0.01*	−0.01	0.95
PCB-187	−0.21	0.08	0.02	0.89
∑PCBs	−0.38	< 0.01*	−0.08	0.49
∑POPs	−0.25	0.04*	−0.06	0.64

* Estimate is statistically significant at α≤0.05.

**Table 4:** Results for linear regressions, adjusted for age and smoking, of POP concentrations (log-transformed) on %5-mC.^a^

	*Alu*		LINE-1	
Chemical	β-Estimate	*p*-Value	β-Estimate	*p*-Value
DDT	−0.26	0.01*	0.06	0.87
DDE	−0.38	0.01*	−0.02	0.97
β-HCH	−0.48	< 0.01*	−0.90	0.09
Hexachlorobenzene	0.06	0.72	−0.72	0.19
Chlordane	−0.21	0.11	−0.46	0.27
*cis*-Chlordane	0.02	0.91	−0.01	0.99
Oxychlordane	−0.32	< 0.01*	−0.66	0.09
α-Chlordane	−0.75	0.05*	−0.62	0.60
Mirex	−0.27	0.01*	−0.23	0.51
Toxaphene	0.09	0.51	−0.16	0.72
PCB-28	−0.42	< 0.01*	−0.55	0.23
PCB-52	−0.12	0.36	−0.05	0.91
PCB-99	−0.51	< 0.01*	−0.55	0.22
PCB-101	−0.25	0.05*	0.33	0.44
PCB-105	−0.51	< 0.01*	−0.71	0.08
PCB-118	−0.49	< 0.01*	−0.73	0.12
PCB-128	−0.52	< 0.01*	−0.01	0.99
PCB-138	−0.54	< 0.01*	−0.52	0.24
PCB-153	−0.52	< 0.01*	−0.47	0.22
PCB-156	−0.66	< 0.01*	−0.48	0.26
PCB-170	−0.54	< 0.01*	−0.48	0.15
PCB-180	−0.54	< 0.01*	−0.43	0.24
PCB-183	−0.57	< 0.01*	−0.20	0.67
PCB-187	−0.49	< 0.01*	−0.15	0.77
∑PCBs	−0.56	< 0.01*	−0.49	0.23
∑POPs	−0.48	< 0.01*	−0.46	0.32

a Age variable was continuous; smoking variable was continuous, reflecting number of cigarettes per day (or zero if a non-smoker).

* Estimate is statistically significant at α≤0.05.

## References

[b1-ehp-116-1547] AMAP (2002). Arctic Pollution 2002.

[b2-ehp-116-1547] Anway MD, Leathers C, Skinner MK (2006). Endocrine disruptor vinclozolin induced epigenetic transgenerational adult-onset disease. Endocrinology.

[b3-ehp-116-1547] Anway MD, Skinner MK (2006). Epigenetic transgenerational actions of endocrine disruptors. Endocrinology.

[b4-ehp-116-1547] Ayotte P, Dewailly E, Ryan J, Bruneau S, Lebel G (1997). PCBs and dioxin-like compounds in plasma of adult Inuit living in Nunavik (Arctic Quebec). Chemosphere.

[b5-ehp-116-1547] Balada E, Ordi-Ros J, Vilardell-Tarres M (2007). DNA methylation and systemic lupus erythematosus. Ann N Y Acad Sci.

[b6-ehp-116-1547] Bollati V, Baccarelli A, Hou L, Nonzini M, Fustioni S, Cavallo D (2007). Changes in DNA methylation patterns in subjects exposed to low-dose benzene. Cancer Res.

[b7-ehp-116-1547] Brunaud L, Alberto J, Ayav A, Gerard P, Namour F, Antunes L (2003). Effects of vitamin B12 and folate deficiencies on DNA methylation and carcinogenesis in rat liver. Clin Chem Lab Med.

[b8-ehp-116-1547] Butler Walker J, Seddon L, McMullen E, Houseman J, Tofflemire K, Corriveau A (2003). Organochlorine levels in maternal and umbilical cord blood plasma in Arctic Canada. Sci Total Environ.

[b9-ehp-116-1547] Chang H, Anway M, Rekow S, Skinner MK (2006). Trans-generational epigenetic imprinting of the male germline by endocrine disruptor exposure during gonadal exposure during gonadal sex determination. Endocrinology.

[b10-ehp-116-1547] Cocco P, Fadda D, Ibba A, Melis M, Tocco MG, Atzeri S (2005). Reproductive outcomes in DDT applicators. Environ Res.

[b11-ehp-116-1547] Colt J, Severson R, Lubin J, Rothman N, Camann D, Davis S (2005). Organochlorines in carpet dust and non-Hodgkin lymphoma. Epidemiology.

[b12-ehp-116-1547] Danish Environmental Protection Agency (1997). AMAP Greenland 1994–1996. Environmental Project No. 356.

[b13-ehp-116-1547] Deutch B, Hansen JC (2000). High human plasma levels of organochlorine compounds in Greenland. Danish Medical Bulletin.

[b14-ehp-116-1547] Dewailly E, Mulvad G, Pedersen H, Ayotte P, Demers P, Weber J (1999). Concentration of organochlorines in human brain, liver, and adipose tissue autopsy samples from Greenland. Environ Health Perspect.

[b15-ehp-116-1547] Dupont J, Tost J, Jammes H, Gut I (2004). De novo quantitative bisulfite sequencing using the pyrosequencing technology. Anal Biochem.

[b16-ehp-116-1547] Engel L, Lan Q, Rothman N (2007). Polychlorinated biphenyls and non-Hodgkin lymphoma. Cancer Epidemiol Biomarkers Prev.

[b17-ehp-116-1547] Fenech M (2001). The role of folic acid and vitamin B12 in genomic stability of human cells. Mutat Res.

[b18-ehp-116-1547] Fraga MF, Agrelo R, Esteller M (2007). Cross-talk between aging and cancer: the epigenetic language. Ann N Y Acad Sci.

[b19-ehp-116-1547] Furniss C, Marsit C, Houseman E, Eddy K, Kelsey K (2008). Line region hypomethylation is associated with lifestyle and differs by human papillomavirus status in head and neck squamous cell carcinomas. Cancer Epidemiol Biomarkers Prev.

[b20-ehp-116-1547] Gonzalgo M, Jones PA (1997). Rapid quantitation of methylation differences at specific sites using methylation-sensitive single nucleotide primer extension (Ms-SNuPE). Nucleic Acids Res.

[b21-ehp-116-1547] Hardell L, Carlberg M, Hardell K, Bjornforth H, Wickbom G, Ionescu M (2007). Decreased survival in pancreatic cancer patients with high concentrations of organ-ochlorines in adipose tissue. Biomed Pharmacother.

[b22-ehp-116-1547] Hoppin J, Tolbert P, Holly E, Brock J, Korrick S, Altshul L (2000). Pancreatic cancer and serum organochlorine levels. Cancer Epidemiol Biomarkers Prev.

[b23-ehp-116-1547] Jones PA, Baylin SB (2002). The fundamental role of epigenetic events in cancer. Nat Rev Genet.

[b24-ehp-116-1547] Kakeyama M, Tohyama C (2003). Developmental neurotoxicity of dioxin and its related compounds. Ind Health.

[b25-ehp-116-1547] Kang SC, Lee BM (2005). DNA methylation of estrogen receptor alpha gene by phthalates. J Toxicol Environ Health A.

[b26-ehp-116-1547] Kazazian H, Goodier J (2002). LINE drive, retrotransposition and genome instability. Cell.

[b27-ehp-116-1547] Korrick SA, Chen C, Damokosh AI, Ni J, Liu X, Cho SI (2001). Association of DDT with spontaneous abortion: a case-control study. Ann Epidemiol.

[b28-ehp-116-1547] Li T, Schmid CW (2001). Differential stress induction of individual Alu loci: implications for transcription and retrotransposition. Gene.

[b29-ehp-116-1547] Liu L, Wylie RC, Andrews LG, Tollefsbol TO (2003). Aging, cancer and nutrition: the DNA methylation connection. Mech Ageing Dev.

[b30-ehp-116-1547] Longnecker MP, Daniels JL (2001). Environmental contaminants as etiologic factors for diabetes. Environ Health Perspect.

[b31-ehp-116-1547] Longnecker MP, Klebanoff MA, Dunson DB, Guo X, Chen Z, Zhou H (2005). Maternal serum level of the DDT metabolite DDE in relation to fetal loss in previous pregnancies. Environ Res.

[b32-ehp-116-1547] Patterson D, Isaacs S, Alexander L, Turner W, Hampton L, Bernert H (1991). Method 6: Determination of specific polychlorinated dibenzo-dioxins and dibenzo-furans in blood and adipose tissue by isotope dilution-high-resolution mass spectrometry. IARC Sci Publ.

[b33-ehp-116-1547] Perrin D, Ballestar E, Fraga MF, Frappart L, Esteller M, Guerin JF (2007). Specific hypermethylation of LINE-1 elements during abnormal overgrowth and differentiation of human placenta. Oncogene.

[b34-ehp-116-1547] Pflieger-Bruss S, Schuppe HC, Schill WB (2004). The male reproductive system and its susceptibility to endocrine disrupting chemicals. Andrologia.

[b35-ehp-116-1547] Porta M, Malats N, Jariod M, Grimalt J, Rifa J, Carrato A (1999). Serum concentrations of organochlorine compounds and K-ras mutations in exocrine pancreatic cancer. PANKRAS II Study Group. Lancet.

[b36-ehp-116-1547] Powell JJ, Van de Water J, Gershwin ME (1999). Evidence for the role of environmental agents in the initiation or progression of autoimmune conditions. Environ Health Perspect.

[b37-ehp-116-1547] Pyathilake C, Johanning G (2002). Cellular vitamins, DNA methylation and cancer risk. J Nutr.

[b38-ehp-116-1547] Ritchie J, Vial S, Fuortes L, Guo H, Reedy V, Smith E (2003). Organochlorines and risk of prostate cancer. J Occup Environ Med.

[b39-ehp-116-1547] Rusiecki J, Matthews A, Sturgeon S, Sinha R, Pellizzari E, Zheng T, Baris D (2005). A correlation study of organochlorine levels in serum, breast adipose tissue, and gluteal adipose tissue among breast cancer cases in India. Cancer Epidemiol Biomarkers Prev.

[b40-ehp-116-1547] Salazar-Garcia F, Gallardo-Diaz E, Ceron-Mireles P, Loomis D, Borja-Aburto VH (2004). Reproductive effects of occupational DDT exposure among male malaria control workers. Environ Health Perspect.

[b41-ehp-116-1547] Schulz WA (2006). L1 retrotransposons in human cancers. J Biomed Biotechnol.

[b42-ehp-116-1547] Seegal RF (1996). Epidemiological and laboratory evidence of PCB-induced neurotoxicity. Crit Rev Toxicol.

[b43-ehp-116-1547] Smith IM, Mydlarz WK, Mithani SK, Califano JA (2007). DNA global hypomethylation in squamous cell head and neck cancer associated with smoking, alcohol consumption and stage. Int J Cancer.

[b44-ehp-116-1547] Smith SS, Crocitto L (1999). DNA methylation in eukaryotic chromosome stability revisited:DNA methyltransferase in the management of DNA conformation space. Mol Carcinog.

[b45-ehp-116-1547] Tabb M, Blumberg B (2006). New modes of action for endocrine-disrupting chemicals. Mol Endocrinol.

[b46-ehp-116-1547] Tilson HA, Kodavanti PR (1998). The neurotoxicity of polychlorinated biphenyls. Neurotoxicology.

[b47-ehp-116-1547] Van Oostdam J, Tremblay N (2003). Biological monitoring: human tissue levels of environmental contaminants. AMAP Assessment 2002: Human Health in the Arctic (Arctic Monitoring and Assessment Program, eds).

[b48-ehp-116-1547] Venners SA, Korrick S, Xu X, Chen C, Guang W, Huang A (2005). Preconception serum DDT and pregnancy loss: a prospective study using a biomarker of pregnancy. Am J Epidemiol.

[b49-ehp-116-1547] Wilson AS, Power BE, Molloy PL (2007). DNA hypomethylation and human diseases. Biochim Biophys Acta.

[b50-ehp-116-1547] Winneke G, Walkowiak J, Lilienthal H (2002). PCB-induced neurodevelopmental toxicity in human infants and its potential mediation by endocrine dysfunction. Toxicology.

[b51-ehp-116-1547] Yang A, Estecio M, Doshi K, Kondo Y, Tajara E, Issa J (2004). A simple method for estimating global DNA methylation using bisulfite PCR of repetitive DNA elements. Nucleic Acids Res.

